# The Interaction between Vergence and Accommodation Cues in the Assessment of Fusional Vergence Range

**DOI:** 10.3390/life14091185

**Published:** 2024-09-19

**Authors:** Marc Argilés, Genis Cardona

**Affiliations:** 1 Departament d’Òptica i Optometria (DOO), Universitat Politècnica de Catalunya (UPC), Campus de Terrassa, Edifici TR8, C.Violinista Vellsolà, 37, 08222 Terrassa, Spain; 2 Centre for Sensors, Instruments and Systems Development (CD6), Universitat Politècnica de Catalunya (UPC), 08222 Terrassa, Spain; 3 Applied Optics and Image Processing Group (GOAPI), School of Optics and Optometry of Terrassa, Universitat Politècnica de Catalunya (UPC), 08222 Terrassa, Spain

**Keywords:** fusional vergence range, accommodation, vergence, proximal vergence

## Abstract

Fusional vergence range tests are commonly used in optometric practice. The aim of this study was to investigate the possible contribution of CA/C, AC/A, and proximal cues (PCT) to the magnitude and presence of blur and recovery during the measurement of fusional vergence ranges and to determine whether the occurrence of blur is influenced by these vergence and accommodation cues. A total of 27 participants with normal binocular vision were included and AC/A, CA/C, and PCT ratios were evaluated. Blur, break, and recovery values in convergence and divergence were assessed with base-out and base-in prisms, respectively. No statistical correlations were found between AC/A, CA/C, and PCT ratios and the magnitude of blur, break, and recovery values in neither far, near, convergence, nor divergence testing conditions. However, better near point of convergence values were related to higher break values in convergence at far distances, but not at near distances. In addition, for convergence and far distance, a statistical difference was found between groups reporting and not reporting blur in AC/A stimulus and PCT ratios. The present results cannot confirm whether vergence and accommodation cues, such as AC/A, CA/C, and PCT ratios, may play an active role during the assessment of fusional vergence range.

## 1. Introduction

Clinical evaluation of horizontal fusional vergence range is an important part of optometric testing. It can be evaluated for horizontal vergence, stimulating the range of convergence with a base-out (BO) prism, or for divergence, with a base-in (BI) prism, at both near (usually at 0.4 meters (m)) and far (usually at 6.0 m) distances [1,2,3]. The vertical fusional vergence range may also be evaluated, especially in the presence of vertical phoria. This study focuses on the horizontal fusional vergence range, which will be referred to simply as the fusional vergence range. 

The balance between the magnitude of fusional vergence range and phoria determines the risk of increased visual symptoms at both near and far distances [4,5,6,7,8,9,10]. Clinical criteria to determine this balance have been explored and are commonly used in clinical practice for exophoria (Sheard criteria) and esophoria (Percival criteria) [11]. In addition, at each distance and vergence demand (BO and BI), the typical recording points for fusional vergence range with increasing vergence demand are blur (the patient reports blur of the presented visual stimulus) and break (the patient reports double vision). At this point, the clinician decreases the vergence demand, and the patient reports the recovery point (single and clear vision). Normative values for vergence range have been studied and published to guide clinicians in the management of their patients [6,7].

**Figure 1:**
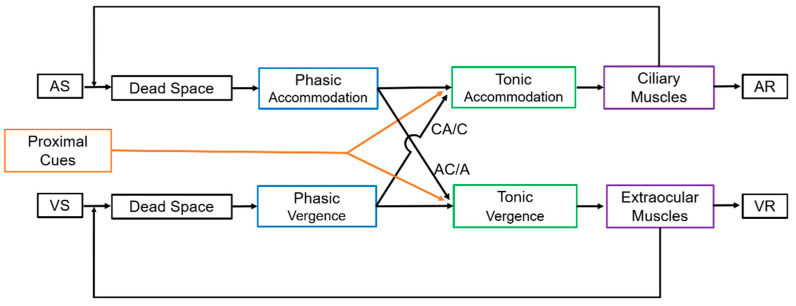
Schematic of the dual-static accommodation and vergence system. This model is based on the classical work of Hung, Ciuffreda, and Rosenfield [15]. For a viewing distance of 0.4 m, the accommodative stimulus (AS) is 2.50 D, and for a standard pupillary distance of 6.0 cm, the vergence stimulus (VS) is 6 × 2.5 = 15 prism diopters (∆). Some authors prefer to use a meter angle (MA), which represents the reciprocal distance for the stimulus (MA = 1/distance (m)). The ocular convergence in prism diopters is the interpupillary distance (IPD) multiplied by MA. Thus, for example, for an IPD of 6 cm and a stimulus at 0.4 m, the ocular convergence is 6 × 2.5 = 15 ∆. Dead Space for accommodation corresponds to the depth of focus and for vergence to the Panum’s area. The phasic and tonic accommodation for each system is represented, as well as the negative loops. The VCG gain is shown as the flow arrow between VS and VR. Proximal cues contribute to the dual system. AS = Accommodative Stimulus, VS = Vergence Stimulus, AR = Accommodative Response, VR = Vergence Response.

Interestingly, the physiological basis for the magnitude and presence of blur and recovery during the evaluation of fusional vergence is not fully understood. The magnitude of the break point has a central mechanism component. Some authors have referred to a vergence controller gain (VCG) and to the extraocular muscle function [12,13,14,15]. The vergence and accommodation interaction, which is represented by some components such as the AC/A ratio, CA/C ratio, and proximal vergence may influence the final output for vergence and accommodation response. Theoretical models have been studied to explain this interaction, which is represented by a negative feedback (loop) for both the vergence and accommodative systems, and includes phasic (fast) and tonic (slow) components (Figure 1). The AC/A and CA/C ratio represents the cross-link between those systems, with proximal vergence and accommodation cues providing a smaller contribution to the final add-up visual response. 

The AC/A ratio is commonly measured in clinical practice, and can be evaluated using heterophoria or the gradient technique [1,2]. This value shall be employed to determine the best treatment option for each patient and for the diagnosis of binocular anomalies such as convergence insufficiency, which has a low AC/A ratio, or excess of divergence, where this ratio is abnormally high [16,17]. Conversely, the CA/C ratio is not commonly evaluated in daily clinical routines, although some studies have shown its importance for the diagnosis of binocular and accommodative anomalies [18,19,20,21,22,23]. This ratio is often overlooked. Indeed, to evaluate the sole contribution of vergence to the accommodation response, the accommodative system must be static (or not responsive) during the test, which is commonly known as “open-loop” in the negative feedback (Figure 1). For the AC/A ratio, it is easier to open-loop the vergence system using a prism to dissociate and obtain double vision (Von Graefe technique), or to assess it monocularly (with the Cover Test or Maddox Rod methods). 

Some studies have used a Difference of Gaussians (DoG) stimulus to open-loop the accommodative system, with good agreement between stimulus and response CA/C ratios [24]. One study found that the AC/A gradient, rather than the calculated AC/A, correlated better with the CA/C ratio [25], although it must be noted that in clinical practice the stimulus rather than the response is commonly determined for both CA/C and AC/A ratios. Sweeney et al. (2014) found a good agreement between stimulus and response for both the AC/A and CA/C ratios, although poorer for CA/C, and noted that the results of the CA/C assessment displayed high variability, thus justifying the fact that this ratio is not often determined [26]. Indeed, the clinician needs experience to detect minimal changes in accommodation (using the MEM or NOTT techniques to evaluate the accommodative error). In addition, CA/C values are very small, ranging from 0.040 D/∆to 0.580 D/∆, with a mean of 0.080 D/∆ [3,20].

Hence, when evaluating fusional vergence, an increase in vergence demand is added, and the amount of prism in the vergence cross-links to the accommodation response due to the effective CA/C ratio. For instance, with a BI prism, the slow mechanism adapts to the new vergence demand when surpassing the accommodative lag (usually +0.50 D), and the overall contribution of the CA/C could determine the magnitude of the blur and recovery points. In the case of convergence, with increasing vergence demand, the effective contribution of the CA/C ratio determines the ability of the patient to increase the accommodative system response to maintain single vision. Two assumptions, untested until now, may be put forward at this point: the larger the CA/C ratio, the more likely a blur will be reported; and the lower this ratio, the more likely that break is reported without a previous blur [27]. 

In addition, patients can use different cues (blur or disparity-driven) to adjust their visual system. A series of studies conducted by Horwood and Riddell revealed that in cases of esotropia and exotropia, near responses to cues differed from those in a non-strabismic population [25,28,29,30,31,32], and one study concluded that proximal vergence contributes to the control of intermittent exotropia [33]. Likewise, the actual mechanism influenced by the CA/C and AC/A ratios and proximal cues, which determines the magnitude or presence of break and recovery during the assessment of fusional vergence ranges, in either BI or BO conditions, remains poorly understood.

The interplay between the AC/A, CA/C, and proximal vergence cues and the fusional vergence range is complex and essential for maintaining binocular vision. Understanding these relationships is crucial for clinicians when diagnosing and treating binocular vision disorders. Therefore, the aim of this study was to investigate the possible contribution of CA/C, AC/A, and proximal cues to the magnitude and presence of blur and recovery when using BO and BI prisms to determine the fusional vergence range. In addition, it explores whether the occurrence of blur is influenced by high or low cross-link ratios.

## 2. Materials and Methods

### 2.1. Participants Selection

Participants between 18 and 25 years, all university students, were recruited for the study. No random sampling was used in this research. Subjects willing to participate in the study were recruited by order of attendance following a complete optometric examination to determine whether they satisfied the inclusion and exclusion criteria. The inclusion criteria were visual acuity at far and near distances equal or superior to 20/20, and stereoacuity, as measured with the Random Dot test, with both random points and lateral disparity, better than 40 s of arc. Participants with any ocular pathology were excluded, as were those with systemic pathologies or taking medications that could affect vision. All participants needed to fulfill the Sheard criteria in case of exophoria and the Percival criteria in case of esophoria, and display normal values of near point of convergence (NPC) and amplitude of accommodation (AA), with their habitual correction. All participants agreed to participate in the study and signed an informed consent after the nature of the investigation was explained to them. This study was approved by the Institutional Review Board of the UPC (06/2023).

### 2.2. Fusional Vergence Range, Heterophoria, Near Point of Convergence, and Amplitude of Accommodation

All tests were performed in the same order, but taking into account the possible influence of vergence adaptation when determining fusional vergence range. Dissociate phorias at far (6 m) and near (0.4 m) distances were evaluated with the Von Graefe method. Each measure was repeated 3 times, and the median value was calculated. Fusional vergence ranges were measured at distance and near with a phoropter. Patients were particularly instructed to report the first occurrence of blur, break, and recovery conditions. The fusional vergence range was determined starting with divergence at far and near distances, followed by convergence at far and near distances. Near point of convergence was measured with the Royal Air Force (RAF) rule, and breakup values (double vision) were recorded. Monocular amplitude of accommodation was measured in both eyes with the push-up Donder’s technique, using the same RAF rule. We used the minimum amplitude of accommodation of Hofstetter to calculate the threshold for normal amplitude of accommodation [1,2,3].

### 2.3. CA/C Stimulus Ratio

The CA/C ratio was measured using the method described by Tsuetaki and Schor [24], traditionally used in clinical practice. Briefly, a Difference of Gaussians stimulus, included in the reverse of a Wesson chart (Bernell Corporation, Mishawaka, IN, USA), was used for this purpose, which has been shown to display good clinical repeatability. This image subtended 21 degrees of visual angle at 0.4 m. Accommodative error was measured using the modified NOTT technique by calculating the accommodative lag (in D) with and without a 6 Δ BO prism. The CA/C ratio was then calculated as the difference between accommodative errors in both conditions, divided by 6 (expressed in D/Δ units).

### 2.4. AC/A Stimulus Ratio

Interpupillary distance (IPD) was measured with an auto refractometer. The AC/A stimulus ratio was calculated as described in the literature [1,2]. The AC/A stimulus gradient was measured using the Thorington modified test and the Maddox rod. Phoria was measured with and without +2.00 D binocular addition lenses. Each phoria was measured three times and the median value was obtained. The AC/A stimulus gradient ratio was then calculated as the difference in phorias in absolute value, divided by 2 (expressed in Δ/D units).

### 2.5. Proximal Cues (PCT Ratio)

Inputs for proximity in the vergence and accommodative system may include static cues, such as relative size, overlap, perspective, shading, and texture gradients, and dynamic cues, such as motion parallax and loom. Other non-visual cues related to stimulus position may include proprioceptive information such as touch and memory about object distance [15,34,35,36]. Proximal vergence can be expressed as the proximal convergence to testing distance ratio (PCT), that is, the percentage contribution of proximal vergence to the overall vergence response [37,38], and can be calculated from the difference between the AC/A stimulus and gradient ratios [37]. This method was used to calculate the PCT ratio in the present study.

### 2.6. Data Analysis

Data distribution was first analyzed using the Shapiro–Wilk test. Parametric data were expressed as mean and standard deviation (SD) and nonparametric data as median and range or interquartile range (IQR). To study the statistical significance of differences between the two groups, the paired or unpaired *t*-test was employed for parametric data, and the Wilcoxon’s rank test or the Mann–Whitney U test for paired or unpaired non-parametric data, respectively, or when the sample size was under 10, irrespective of normality. Parametric ANOVA or the non-parametric Kruskal–Wallis test were used when three or more groups were compared. For correlation analysis, the Pearson or Spearman correlation tests were used, according to data normality. The IBM SPSS Statistics for Windows, Version 27.0 (Armonk, NY, USA: IBM Corp) was used for data analysis and the G*Power software, Version 3.1, was employed to calculate the post hoc sample size [39]. A *p*-value < 0.05 denoted statistical significance.

## 3. Results

### 3.1. Participants and Descriptive Values

A total of 35 subjects were screened, of whom 27 satisfied the inclusion and exclusion criteria and were finally included in the study, with a mean age ± SD of 21.3 ± 1.4 years, ranging from 18 to 24 years, and 57% were female. The mean refractive spherical error was −0.50 ± 1.29 D for the right eye, ranging from −4.00 D to +3.50 D, and −0.56 ± 1.32 D for the left eye, ranging from −4.00 D to +3.25 D. Astigmatism values were 0.36 ± 0.67 D, ranging from 0.00 D to 3.25 D, and 0.40 ± 0.71 D, ranging from 0.00 D to 3.50 D, for the right and left eyes, respectively. Median and IQR values were 4.00 cm (4.00, 6.00) for NPC; 13.00 D (12.00, 20.00) for AA; 4.40 ∆/D (3.30, 5.40) for the AC/A stimulus; 1.00 ∆/D (1.00, 2.00) for the AC/A gradient; 3.30 ∆/D (2.00, 4.00) for the PCT ratio; and 0.030 D/∆ (0.025, 0.041) for the CA/C ratio. Descriptive values for fusional vergence ranges are shown in Table 1.

### 3.2. Incidence of Blur Reporting during Fusional Vergence Test

At far, 20 participants reported blur (74.1%) during BO testing, and 10 (37.0%) during BI. At near distances, 10 participants (37.0%) reported blur during BO, and 15 participants (55.5%) during BI. At far and near distances, 6 (22.2%) and 8 (29.6%) participants, respectively, reported blur at both BI and BO (Table 2).

### 3.3. Correlation between CA/C and AC/A

The CA/C ratio did not show statistical significant correlations with the AC/A stimulus ratio (rho(27) = 0.34, *p* = 0.084), nor with the AC/A gradient ratio (rho(27) = −0.15, *p* = 0.461). The AC/A stimulus and AC/A gradient ratio were not significantly correlated (rho(27) = 0.13, *p* = 0.514).

### 3.4. Correlations between CA/C, AC/A, and PCT with Break and Recovery Values

At far and divergence, no statistically significant correlations were found between CA/C ratio values and the points of blur (rho(10) = 0.31, *p* = 0.409), break (rho(27) = 0.00, *p* = 0.99), and recovery (rho(27) = −0.06, *p* = 0.765). Similar results were found in convergence conditions for blur (rho(20) = 0.30, *p* = 0.187), break (rho(27) = 0.28, *p* = 0.211), and recovery (rho(27) = 0.22, *p* = 0.273) values. Neither the AC/A nor the PCT ratios presented any statistical correlation with the divergence and convergence blur, break, and recovery values (all *p* > 0.100). 

At near distances, the CA/C ratio was not significantly correlated with the divergence blur (rho(15) = 0.11, *p* = 0.681), break (rho(27) = −0.07, *p* = 0.725) and recovery (rho(27) = 0.16, *p* = 0.405) values, nor with the convergence blur (rho(10) = 0.07, *p* = 0.833), break (rho(27) = 0.07, *p* = 0.714), and recovery (rho(27) = 0.07, *p* = 0.833) values.

**Figure 2:**
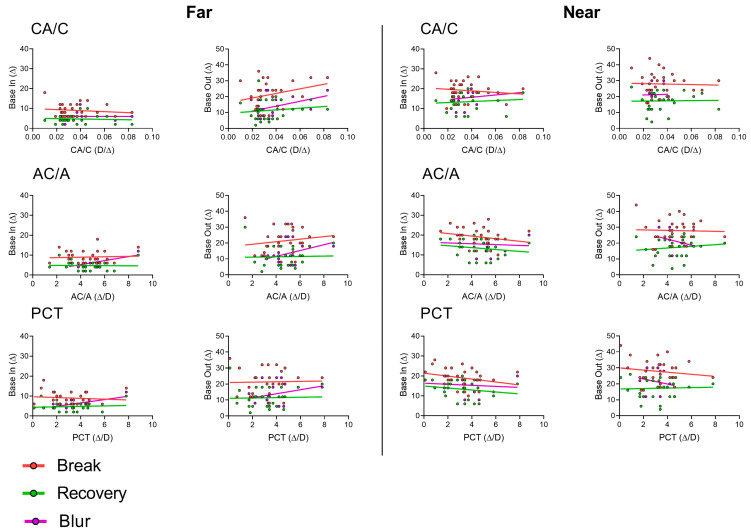
Correlations between CA/C (top), AC/A (middle), and PCT (below) and values of break (red), recovery (green), and blur (purple) at far (6 m) and near (0.4 m) distances in convergence (Base Out) and divergence (Base In) conditions.

A statistically significant moderate negative correlation was found between the NPC and break point values at far (rho(27) = −0.43, *p* = 0.025) in convergence conditions. No further correlations were found between NPC and AA values and break and recovery point values at far or near in convergence or divergence conditions. Figure 2 displays all the correlations between CA/C, AC/A, and PCT values, at far and near distances, and break, recovery, and blur values for both convergence (BO) and divergence (BI) conditions.

### 3.5. AC/A, CA/C, and PCT Values and the Occurrence of Blur

As noted above, at far distance and divergence conditions, 10 participants reported blur. No statistically significant differences were found between subjects reporting and not reporting blur in the AC/A stimulus ratio, *p* = 0.824, AC/A gradient ratio, *p* = 0.863, PCT ratio, *p* = 0.863, and CA/C ratio, *p* = 0.359.

For convergence and far distance, 20 participants reported blur. Statistical differences were found between subjects reporting and not reporting blur in the AC/A stimulus ratio, *p* = 0.033, and PCT ratio, *p* = 0.005, but not in the AC/A gradient ratio, *p* = 0.299, nor in the CA/C ratio, *p* = 0.135. Overall, higher AC/A stimulus and PCT ratios were found in participants who did not report blur, with a difference in medians of 2.00 ∆/D in both parameters.

For near distance and divergence conditions, 15 participants reported blur. No statistically significant differences were found between groups and AC/A stimulus ratio, *p* = 0.494, AC/A gradient ratio, *p* = 0.628, PCT ratio, *p* = 0.328, and CA/C ratio, *p* = 0.510. Finally, at near and convergence conditions, 10 participants reported blur, with no statistical differences between groups in AC/A stimulus ratio, *p* = 0.744, AC/A gradient ratio, *p* = 0.824, PCT ratio, *p* = 0.615, and CA/C ratio, *p* = 0.451.

## 4. Discussion

The aim of this study was to investigate whether independent AC/A, CA/C, and PCT ratios contribute to the magnitude of break and recovery values when assessing the fusional vergence range, and whether the occurrence of blur is influenced by these ratios as a result of the cross-links between the vergence and accommodation systems. In a theoretical, static model, these factors act as cues that contribute to the vergence and accommodative response; however, to the best of our knowledge, the possible contribution of these ratios to the fusional vergence range has not been studied.

The theoretical hypotheses for the contribution of AC/A and CA/C ratios are as follows: with the introduction of BI prisms, patients need to adjust (relax) their accommodative system while increasing divergence, whereas with BO prisms, the accommodative system needs to adjust (activate) while increasing convergence. The effective contribution of both ratios can determine the magnitude of breaking and recovery when assessing the fusional vergence range. In addition, the higher the CA/C ratio, the more likely blur will be reported [27]. The possible contribution of the PCT ratio has not been studied.

The median of AC/A stimulus was 4.40 ∆/, IQR (3.30,5.40), which is the normal value according to the literature [40,41]. The values of the AC/A gradient described in this study were also in agreement with previous research [25]. Albeit this parameter is commonly assessed in clinical practice, its range of normality exhibits high variability depending on the method used to evaluate near and far phoria [42,43].

The present study found a median CA/C ratio of 0.030 D/∆ and IQR (0.025,0.041) with the method proposed by Tsuetaki and Schor [24], who found a mean of 0.050 D/∆. Scheiman and Wick indicated a normal CA/C ratio of approximately 0.080 D/∆ [3], which is close to that reported in other studies [26], although other authors observed a range of normality from 0.040 to 0.220 D/∆ [20]. While some studies have shown that the CA/C ratio could be a clinically useful measure for the diagnosis of binocular and accommodative anomalies [19,21,22], there is a lack of normative values using the same methodology available in common clinical testing. In addition, the CA/C ratio has been found to be influenced by age [23]; therefore, this factor should be considered when comparing normative values of different populations.

The present findings revealed that the closer the NPC, the greater the break magnitude with BO prisms at far distances but not at near distances. These findings suggest that near vision involves a much more complex interaction of the vergence and accommodation systems than far vision. For convergence and far distance, a statistical difference was found in the AC/A stimulus and PCT ratios between groups reporting and not reporting blur, which highlights the role of AC/A in convergence: the higher the AC/A ratio, the lower the possibility of reporting blur. This can be explained by the fact that a high AC/A ratio and better proximal cues can compensate for the vergence and accommodation interactions while increasing the convergence demand, increasing the ability to detect blur at far distances.

This study did not find any correlation between the CA/C and AC/A stimulus ratios, in contrast with previous research authors [25], although measurement procedures for the determination of the CA/C ratio were different. Finally, the PCT values have been seldom investigated [37,38], with only one study analyzing the differences between measurement methods [38]. However, a recent study found that proximal cues contribute significantly to the control of intermittent exotropia and divergence excess [33], as these patients tend to have a high AC/A ratio [3].

No statistical correlations were found between AC/A, CA/C, and PCT ratios and the magnitude of blur, break, and recovery values in neither far, near, BI, nor BO testing conditions. These results are in agreement with the investigations conducted by Horwood et al. [28,31,32], which showed a different use of blur, vergence, and proximity cues among individuals, and an influence of factors such as visual experience and genetic factors. While it is normal to report blur at far distances [6], reporting blur at near distances is highly dependent on each observer. Whereas approximately the same percentage of participants reported blur at near distances in divergent conditions, in convergence, most participants (63.0%) did not report blur. At far distances and convergence, most participants reported blur (74.1%). Therefore, it would seem that each individual exhibits different levels of sensitivity to blur responses that cannot be explained from specific cues alone; however, the higher the AC/A and PCT ratios, the better the ability to detect blur at far vision and convergence. It is known that high or low AC/A and CA/C ratios may lead to binocular dysfunctions, such as convergence insufficiency [1,2,3], which is characterized by high exophoria, low AC/A ratios, high CA/C ratios, and a low convergence fusional vergence range at near distances. In this particular case, the theory indicates that, with a high CA/C ratio, accommodation exceeds vergence, which in turn explains the high exophoria at near distances. Thus, the present findings may be of relevance for individuals with low AC/A ratios and convergence insufficiency, a dysfunction that may be improved with vision therapy [44]. Future studies could assist in explaining how individuals use different cues to adjust their visual system while assessing fusional vergence range. Moreover, although the clinical evaluation of the PCT ratio may contribute to understanding clinical outcomes in far and convergence, the actual impact of this ratio in other visual demands, such as near vision and divergence, remains elusive.

This study is not devoid of limitations. Thus, it may be acknowledged that the current sample size (n = 27) could not be sufficient to reach solid conclusions. Indeed, a post hoc analysis using G*Power [39] to calculate the sample size necessary to detect a difference of 0.010 D/∆ in the CA/C ratio evidenced that a minimum sample size of 120 was needed to achieve 95% power. Therefore, the present findings must be interpreted with caution, given the possibility of Type II statistical error, that is, of failing to detect significant intergroup differences due to insufficient sample size, and the high variability in the methods of measuring the CA/C ratio, and consequent range of normative values, reported in the literature. Other published research studying the interactions of CA/C and AC/A ratios used a sample of n = 25 [26], n = 16 [19], n = 27 [25], n = 26 [20], and even 10 participants to evaluate the change in these parameters obtained with vision therapy [18]. These factors prevent verifying the initial assumptions of this study. 

Notwithstanding these limitations, this study opens a new research avenue to explore the theoretical assumptions related to the vergence and accommodative interactions underpinning fusional vergence range testing in normal binocular vision, and pave the way for future work comparing these relationships in normal and binocular disorders, such as convergence insufficiency or accommodative insufficiency. 

In conclusion, the evaluation of AC/A, CA/C, and PCT ratios demonstrates high variability among individuals, with implications in clinical practice. The present results cannot confirm whether vergence and accommodation cues, such as AC/A, CA/C, and PCT ratios, are involved in the assessment of the fusional vergence range, particularly in the magnitude of blur, break, and recovery values, nor the actual presence of a blur point. However, these findings highlight that at far distance and convergence conditions, lower NPC and higher AC/A stimulus ratios contribute to a larger magnitude of the break point. These results may be of interest to clinicians when exploring the fusional vergence range, and give support to the recommendation of exploring AC/A, CA/C, and proximal vergence in clinical practice, particularly in patients with convergence insufficiency.

## Figures and Tables

**Table 1:** Descriptive statistics for the results of blur (b), break (B), and recovery (r) values using base-out (BO) and base-in (BI) prisms for the convergence and divergence range, respectively. Median and interquartile range (25% and 75%) values are shown. All units are prism diopters (∆).

	Far (6.0 m)						Near (0.4 m)					
	BI			BO			BI			BO		
	b	B	r	b	B	r	b	B	r	b	B	r
Median	6.00	8.00	4.00	12.00	20.00	12.00	18.00	20.00	14.00	21.00	28.00	18.00
IQR (25%)	4.00	6.00	3.50	8.00	14.00	6.00	12.00	16.00	10.00	12.00	24.00	12.00
IQR (75%)	7.00	12.00	6.00	18.00	30.00	18.00	18.00	22.00	18.00	28.50	32.00	22.00

**Table 2:** Incidence, expressed in number of participants and percentage (%), of blur at far, near, and divergence (BI) and convergence (BO) conditions.

Far (6.0 m)				Near (0.4 m)			
BI		BO		BI		BO	
Blur	No blur	Blur	No blur	Blur	No blur	Blur	No blur
10 37.0%	17 63.0%	20 74.1%	7 25.9%	15 55.5%	12 44.5%	10 37.0%	17 63.0%

## Data Availability

The data that support the findings of this study are available from the corresponding author, M.A., upon reasonable request.

## References

[1] Grosvenor T., Grosvenor T.P. (2007). Primary Care Optometry.

[2] Rosenfield M., Logan N. (2009). Optometry: Science, Techniques and Clinical Management.

[3] Scheiman M., Wick B. (2008). Clinical Management of Binocular Vision: Heterophoric, Accommodative, and Eye Movement Disorders.

[4] Antona B., Barrio A., Barra F., Gonzalez E., Sanchez I. (2008). Repeatability and agreement in the measurement of horizontal fusional vergences. Ophthalmic Physiol. Opt..

[5] Fray K.J. (2017). Fusional amplitudes: Developing testing standards. Strabismus.

[6] Lança C.C., Rowe F.J. (2019). Measurement of fusional vergence: A systematic review. Strabismus.

[7] Palomo Álvarez C., Puell M.C., Sánchez-Ramos C., Villena C. (2006). Normal values of distance heterophoria and fusional vergence ranges and effects of age. Graefes Arch. Clin. Exp. Ophthalmol..

[8] Rovira-Gay C., Mestre C., Argiles M., Vinuela-Navarro V., Pujol J. (2023). Feasibility of measuring fusional vergence amplitudes objectively. PLoS ONE.

[9] Rowe F.J. (2010). Fusional vergence measures and their significance in clinical assessment. Strabismus.

[10] Sreenivasan V., Babinsky E.E., Wu Y., Candy T.R. (2016). Objective measurement of fusional vergence ranges and heterophoria in infants and preschool children. Investig. Opthalmology Vis. Sci..

[11] Moon B.-Y., Kim S.-Y., Yu D.-S. (2020). Receiver operating characteristic curve analysis of clinical signs for screening of convergence insufficiency in young adults. PLoS ONE.

[12] Hung S.S., Fisher A.G., Cermak S.A. (1987). The performance of learning-disabled and normal young men on the test of visual-perceptual skills. Am. J. Occup. Ther..

[13] Hung G.K. (1991). Linear model of accommodation and vergence can account for discrepancies between AC/A measures using the fixation disparity and phoria methods. Ophthalmic Physiol. Opt..

[14] Hung G.K. (1992). Adaptation model of accommodation and vergence. Ophthalmic Physiol. Opt..

[15] Hung G.K., Ciuffreda K.J., Rosenfield M. (1996). Proximal contribution to a linear static model of accommodation and vergence. Ophthalmic Physiol. Opt..

[16] Gwiazda J., Thorn F., Held R. (2005). Accommodation, accommodative convergence, and response AC/A ratios before and at the onset of myopia in children. Optom. Vis. Sci..

[17] Arnoldi K.A., Reynolds J.D. (2006). Diagnosis of pseudo-divergence excess exotropia secondary to high accommodative convergence to accommodation ratio. Am. Orthopt. J..

[18] Brautaset R.L., Jennings A.J.M. (2006). Effects of orthoptic treatment on the CA/C and AC/A ratios in convergence insufficiency. Investig. Opthalmology Vis. Sci..

[19] Fukushima T., Torii M., Ukai K., Wolffsohn J.S., Gilmartin B. (2009). The relationship between CA/C ratio and individual differences in dynamic accommodative responses while viewing stereoscopic images. J. Vis..

[20] Hirani K.J., Firth A.Y. (2009). Convergence accommodation to convergence (CA/C) ratio: Stability with different levels of convergence demand. Br. Ir. Orthopt. J..

[21] Neveu P., Roumes C., Philippe M., Fuchs P., Priot A.-E. (2016). Stereoscopic viewing can induce changes in the CA/C ratio. Investig. Opthalmology Vis. Sci..

[22] Rosenfield M., Gilmartin B. (1988). Assessment of the CA/C ratio in a myopic population. Am. J. Optom. Physiol. Opt..

[23] Rosenfield M., Ciuffreda K.J., Chen H.-W. (1995). Effect of age on the interaction between the AC/A and CA/C ratios. Ophthalmic Physiol. Opt..

[24] Tsuetaki T.K., Schor C.M. (1987). Clinical method for measuring adaptation of tonic accommodation and vergence accommodation. Am. J. Optom. Physiol. Opt..

[25] Horwood A.M., Riddell P.M. (2013). The clinical near gradient stimulus AC/A ratio correlates better with the response CA/C ratio than with the response AC/A ratio. Strabismus.

[26] Sweeney L.E., Seidel D., Day M., Gray L.S. (2014). Quantifying interactions between accommodation and vergence in a binocularly normal population. Vis. Res..

[27] Benjamin W.J. (2006). Borish’s Clinical Refraction.

[28] Horwood A.M., Riddell P.M. (2008). The use of cues to convergence and accommodation in naïve, uninstructed participants. Vis. Res..

[29] Horwood A.M., Riddell P.M. (2012). Decreased accommodation during decompensation of distance exotropia. Br. J. Ophthalmol..

[30] Horwood A.M., Riddell P.M. (2012). Evidence that convergence rather than accommodation controls intermittent distance exotropia. Acta Ophthalmol..

[31] Horwood A.M., Riddell P.M. (2013). Accommodation and vergence response gains to different near cues characterize specific esotropias. Strabismus.

[32] Horwood A.M., Riddell P.M. (2014). Disparity-driven vs blur-driven models of accommodation and convergence in binocular vision and intermittent strabismus. J. AAPOS.

[33] Mestre C., Neupane S., Manh V., Tarczy-Hornoch K., Candy T.R. (2023). Vergence and accommodation responses in the control of intermittent exotropia. Ophthalmic Physiol. Opt..

[34] Joubert C., Bedell H.E. (1990). Proximal vergence and perceived distance. Optom. Vis. Sci..

[35] Wick B. (1985). Clinical factors in proximal vergence. Am. J. Optom. Physiol. Opt..

[36] Wick B., Bedell H.E. (1989). Magnitude and velocity of proximal vergence. Investig. Opthalmology Vis. Sci..

[37] Fogt N., Toole A.J., Rogers D.L. (2016). A review of proximal inputs to the near response. Clin. Exp. Optom..

[38] Fogt N. (2020). Comparisons of proximal vergence measures. Vis. Dev. Rehabil..

[39] Faul F., Erdfelder E., Lang A.-G., Buchner A. (2007). G* Power 3: A flexible statistical power analysis program for the social, behavioral, and biomedical sciences. Behav. Res. Methods.

[40] Murray C., Newsham D. (2018). The normal accommodative convergence/accommodation (AC/A) ratio. J. Binocul. Vis. Ocul. Motil..

[41] Gwiazda J., Grice K., Thorn F. (1999). Response AC/A ratios are elevated in myopic children. Ophthalmic Physiol. Opt..

[42] Bhoola H., Bruce A.S., Atchison D.A. (1995). Validity of clinical measures of the AC/A ratio. Clin. Exp. Optom..

[43] Rainey B.B., Goss D.A., Kidwell M., Feng B. (1998). Reliability of the response AC/A ratio determined using nearpoint autorefraction and simultaneous heterophoria measurement. Clin. Exp. Optom..

[44] Singh N.K., Mani R., Hussaindeen J.R. (2017). Changes in stimulus and response AC/A ratio with vision therapy in Convergence Insufficiency. J. Optom..

